# Primary Endometrial Diffuse Large B-cell Lymphoma: A Rare Disease and Diagnostic Challenge in an Asymptomatic Postmenopausal Woman

**DOI:** 10.7759/cureus.24592

**Published:** 2022-04-29

**Authors:** Iqra Arshad, Muhammad Kafeel

**Affiliations:** 1 Internal Medicine, Lincoln Medical and Mental Health Center, New York, USA; 2 Hematology and Medical Oncology, Flushing Hospital Medical Center, New York, USA

**Keywords:** extranodal diffuse large b-cell lymphoma, ann arbor staging system, hematology oncology, r-chop therapy, extranodal involvement

## Abstract

Primary endometrial lymphomas are rare malignancies because the female genital tract is usually involved as a secondary site. Here, we present a case of primary endometrial lymphoma diagnosed in a 49-year-old postmenopausal female who was referred to hematology/oncology service for the evaluation of incidental findings of malignant-looking cells on a Pap smear followed by a cervical polyp biopsy that was suggestive of high-grade B-cell lymphoma (Ki-67: 80-90%) on routine screening. The baseline laboratory assessment was unremarkable except for hypochromic normocytic anemia. A bone marrow biopsy was performed to rule out primary involvement and revealed no evidence of lymphoma both on morphology and immunophenotyping. Fluorescence in situ hybridization tests were also negative. Repeat endocervical biopsy with more tissue sampling revealed similar findings. Further workup was pursued including an initial staging positron emission tomography-computed tomography (PET-CT) scan that showed a 3.8 × 2.7 cm, with standardized uptake value (SUV)max of 30.4, malignant-appearing mass extending up to the left posterior cervix and an 11 mm left axillary lymph node with SUVmax of 2.9. An excisional biopsy of the axillary node was negative for malignancy and ruled out nodal involvement. A diagnosis of primary endometrial diffuse large B-cell lymphoma was made on biopsy of posterior cervical mass that revealed diffuse infiltration of large lymphoid cells, positive for B-cell markers, namely, B-cell lymphoma 6 (BCL6), paired box 5 (PAX5+), CD20, and CD19 with methoxyisobutyl isonitrile (MIBI) of 100%, and negative for T-cell and mesenchymal markers, namely, CD3, CD45, CD43, CD138, Melan A, S100, and Vimentin. The disease was staged as 1E (one extranodal site) according to the Ann Arbor staging system. The patient achieved remission after receiving four to six cycles of R-CHOP (rituximab, cyclophosphamide, hydroxydaunorubicin, oncovin, and prednisone) therapy. Interval staging PET-CT scans, performed after the second cycle and at the completion of therapy to assess treatment response, were negative for new disease activity in the uterus. The patient remains in clinical remission to date and is on regular follow-up. This case is a good illustration of the fact that the female genital tract can be the primary site for B-cell lymphomas. If such an abnormality is found incidentally on routine screening, it should not be ignored and the patient should be evaluated further to make the definitive diagnosis so that timely management can be offered. Through this case, we also highlight the role of immunohistochemical studies using specific cell markers in ruling out other possibilities that could mimic lymphomas on tissue biopsy as treatment modalities differ.

## Introduction

Primary involvement of the female genital tract by lymphoproliferative disorders, such as non-Hodgkin’s lymphomas, is an extremely rare diagnosis. Uterine and cervical lymphomas account for only 1.5% of extranodal non-Hodgkin’s lymphomas and fewer than 0.5% of all gynecological malignancies [1]. The most common extranodal sites of lymphomas are the gastrointestinal tract and skin. However, the female reproductive system may also be affected, and the uterine cervix is the most rarely involved site [2]. In many such cases, B-cell lymphomas are often found incidentally on routine screening, that is, Papanicolaou (Pap) smear or cervical polyp biopsy [3]. Because of the rarity of primary gynecological lymphomas, a standard treatment approach has not been developed [4]. However, proposed treatment options include surgery, radiation therapy, chemotherapy, or immunochemotherapy with the addition of rituximab to the CHOP (cyclophosphamide, hydroxydaunorubicin, oncovin, and prednisone) regimen, also known as R-CHOP therapy.

## Case presentation

We present the case of a 49-year-old postmenopausal Asian female, a non-smoker and non-drinker with a medical history of type 2 diabetes mellitus, hypertension, and hyperlipidemia, who was referred by her OB/GYN doctor to the hematology/oncology service for the evaluation of an abnormal Pap smear and cervical polyp biopsy report. The Pap smear was positive for malignant-looking cells and negative for human papillomavirus-RNA and other sexually transmitted pathogens, including gonococcus and chlamydia. Pathology of the cervical polyp biopsy was consistent with diffuse infiltration by large lymphoid cells with slightly irregular nuclei; multiple distinct nucleoli and scant cytoplasm; multiple mitotic figures; and cells strongly positive for CD20, B-cell lymphoma (BCL)6, and partially for BCL2. The findings were suggestive of high-grade B-cell lymphoma with Ki-67 of approximately 80-90%. Upon further review of systems, pertinent positive findings included increasing fatigue, tiredness, and anorexia. The patient denied a history of postmenopausal bleeding or any other bleeding activity. She further denied B-symptoms (fevers without infection, drenching night sweats, and unintentional weight loss of >10% of body weight over six months), abdominal pain, and altered bowel and urinary habits. A detailed clinical examination was unremarkable for pallor, generalized lymphadenopathy, and hepatosplenomegaly. The pelvic examination was also unremarkable.

**Figure 1:**
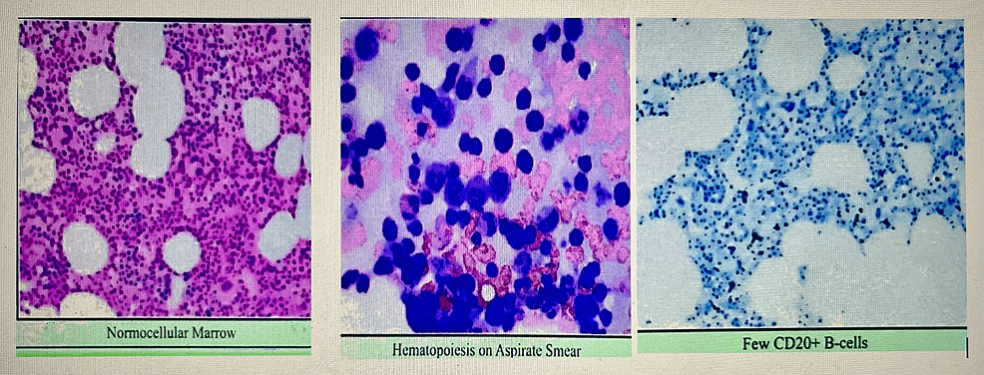
Bone marrow biopsy showing normocellular marrow and few CD20+ B-cells (on the right side).

The baseline laboratory workup was unremarkable except for mild hypochromic normocytic anemia. The initial plan was to send the patient back to OB/GYN service for a repeat endocervical biopsy with more tissue sampling and to schedule her for a bone marrow biopsy. The patient returned for follow-up, and a repeat endocervical biopsy report was suggestive of high-grade lymphoid neoplasm. Bone marrow biopsy showed normocellular marrow with no evidence of involvement by B-cell lymphoma both on morphology and immunophenotyping (Figure 1). Fluorescence in situ hybridization studies revealed normal cytogenetics with no evidence of MYC-break apart rearrangements, BCL2-immunoglobulin heavy chain translocation, t (14;18), and BCL6 (3q27) breakpoint translocations.

**Figure 2:**
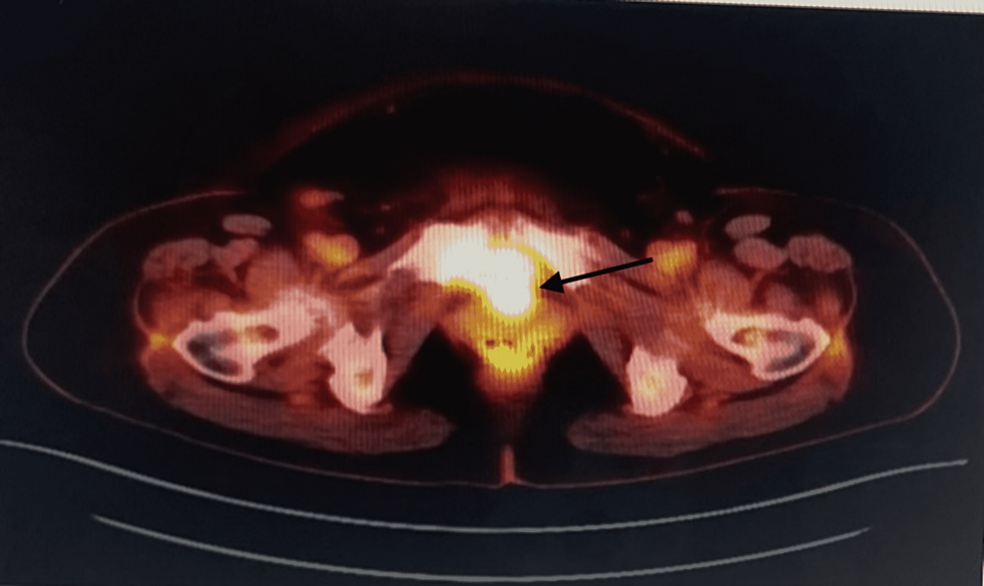
Initial staging PET-CT scan showing a malignant-appearing mass in the left posterior cervix. PET-CT: positron emission tomography-computed tomography

An initial positron emission tomography-computed tomography (PET-CT) scan from the base of the skull to the mid-thigh was performed to look for the primary and to stage the disease. PET-CT scan revealed a 3.8 × 2.7 cm malignant-appearing mass with a standardized uptake value (SUV)max of 30.4, extending from the lower left posterior aspect of the uterus, (Figure 2), an 11 mm left axillary lymph node with fatty hilum, and up to 4 mm thick cortex with SUVmax of 2.9 with few active bilateral neck level two nodes; on the right, SUVmax of 5.1 corresponding to a 6 mm node, and on the left, SUVmax of 4.8 corresponding to a 10 mm node. Endoscopy and colonoscopy were performed as part of the anemia workup and were negative for any significant findings. Because the SUV of the cervical mass was very high, further management options of getting a biopsy of the mass for a definitive diagnosis and axillary lymph node excisional biopsy to rule out nodal involvement were discussed with the patient and her family. The patient agreed to both procedures. GYN oncology and general surgery teams were involved in the patient’s care at this point.

Left axillary lymph node excisional biopsy was suggestive of reactive changes with follicular hyperplasia and was negative for lymphoma. Biopsies were obtained from three different sites of cervical mass. Biopsy findings were consistent with diffuse infiltration by lymphoid cells that were positive for BCL6, paired box 5 (PAX5+), CD20, CD19, with very high methoxyisobutyl isonitrile (MIBI) approaching 100%, and negative for CD3, CD45, CD43, CD138, Melan A, AE1/3, S100, Vimentin, SOX10, and PAX8 on immunophenotyping. The patient was diagnosed with low-grade primary endometrial diffuse large B-cell lymphoma (DLBCL) and the disease was staged as 1E according to the Ann Arbor staging system.

**Figure 3:**
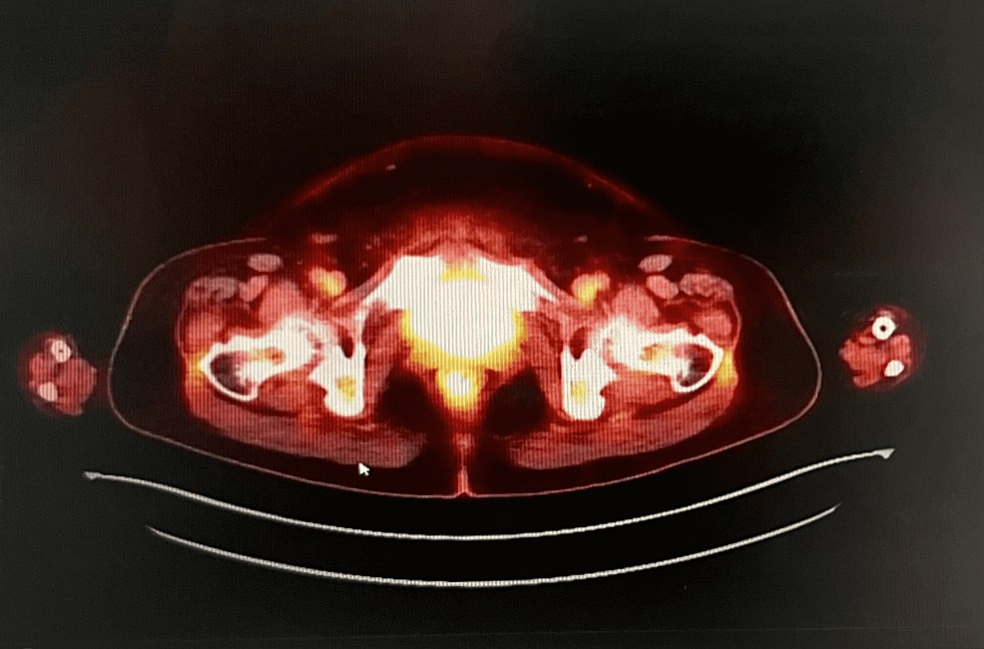
Restaging PET-CT scan at the completion of chemotherapy showing no evidence of any malignant activity in the uterus. PET-CT: positron emission tomography-computed tomography

The patient was scheduled for four to six cycles of R-CHOP therapy. Informed consent was taken and the side effects of chemoimmunotherapy were discussed in detail. Baseline echocardiogram was performed before starting chemotherapy and showed normal ejection fraction. A chemo-port was placed and therapy was started. A restaging PET-CT scan was obtained at the completion of the second cycle to evaluate response to the treatment showed no evidence of any malignant-appearing activity in the uterus. Therapy was continued with good compliance. Patient tolerated the chemotherapy well. The only complications she developed were mucositis, which was managed conservatively, and severe neutropenia without fevers. For her neutropenia, she received granulocyte colony-stimulating factor with intravenous hydration. At the completion of therapy, a repeat PET-CT scan (Figure 3) showed no evidence of malignant-appearing activity in the uterus. Repeat complete blood count after completion of therapy showed normocytic normochromic anemia and resolution of neutropenia. The patient remains in clinical remission to date and is on regular follow-up with the hematology/oncology service.

## Discussion

DLBCLs are lymphoid malignancies accounting for up to 35% of non-Hodgkin’s lymphomas in the adult population of the western world, with a higher incidence in developing nations. In up to 40% of DLBCL cases, the tumor presents at an extranodal site with the gastrointestinal tract being the most commonly involved. Primary involvement of the uterine cavity by lymphoid neoplasm is an extremely rare manifestation of the disease as the female genital tract is most often affected secondarily [5,6]. Primary endometrial lymphomas usually affect postmenopausal women [7,8]. Patients tend to present with abnormal vaginal bleeding such as menorrhagia, postmenopausal bleeding, or abnormal vaginal discharge [9]. However, in our case, the patient did not have any vaginal bleeding and presented with non-specific symptoms. In this case, the abnormality was picked up incidentally on routine screening, and the diagnosis was challenging until it was confirmed on biopsy. Therefore, histopathological evaluation is mandatory for the definitive diagnosis of lymphomas.

Criteria that must be fulfilled for diagnosis of a primary endometrial lymphoma, as proposed by Fox et al., include the following: confinement of the disease to the uterus at the time of the first diagnosis, no identifiable leukemia on a full blood count, no evidence of disease at other sites in the body, and a period of a number of months passing between the identification of a secondary site of involvement and the initial tumor site [6,7,10]. In our case, the tumor was confined to the uterus at the time of diagnosis with no evidence of nodal and bone marrow involvement, thus fulfilling the criteria.

While making the definitive diagnosis of lymphoma from a biopsy report where dys-cohesive large lymphoid cells are found arranged in sheets, it is important to consider a broader differential diagnosis including poorly differentiated carcinomas, malignant mixed Mullerian tumor, high-grade endometrial stromal sarcoma, Ewing sarcoma, amelanotic melanoma, other hematopoietic malignancies such as Burkitt lymphoma, peripheral T-cell lymphoma not otherwise specified, anaplastic large cell lymphoma, as well as myeloid and reactive lymphoid hyperplasia or lymphoma-like lesions. Morphology and immunohistochemical studies using lymphoid, epithelial, and mesenchymal cell markers assist in differentiating these neoplasms. In our case, the tumor cells were strongly positive for B-cell markers and negative for T-cell and other stromal cell markers, excluding all these possible differentials.

As primary endometrial lymphoma is an uncommon diagnosis, extensive studies have not been performed. No standardized treatment has been proposed. In the past, different treatment modalities have been used either alone or in combination, including surgery, radiation therapy, and chemotherapy. The use of chemotherapy combined with radiation was reported by Stroh et al., who reported 16 cases of lymphomas of the cervix, of which 12 received radiation. In this case series, around 90% of patients with low risk factors were disease-free at five years [4].

Cubo et al. noted that, with the passage of time, chemotherapy with or without radiation therapy has become the preferred treatment option compared to previous treatment plans in which surgery was the predominant modality [11]. Furthermore, the addition of the monoclonal antibody, rituximab, to chemotherapeutic agents (cyclophosphamide, doxorubicin, oncovin, and prednisolone, known as R-CHOP therapy), has resulted in increased overall survival for patients with DLBCL [12]. According to previous studies, in some cases, the complete remission rate with immunochemotherapy without surgery is 75% [12]; however, Horning et al. [13] demonstrated an increased 10-year disease-free survival in patients who were treated with combined therapy. Presently, a combined treatment approach is more favored as immuno-chemotherapeutic agents prevent disease relapse and radiation decreases the likelihood of local recurrence [13,14].

## Conclusions

Primary endometrial B-cell lymphoma is a challenging diagnosis because of its rarity, particularly in a patient who is either completely asymptomatic or presents with non-specific symptoms as observed in the presented case. Many times, such abnormalities are just picked up incidentally on routine screening; therefore; a thorough approach is warranted and further investigation should be offered to patients. A broader differential diagnosis should be considered while making the definitive diagnosis as many other pathologies mimic lymphomas. Further, immunohistochemical studies using cell-specific markers help in excluding these possibilities. Timely diagnosis and management are paramount to improving the prognosis. This case also emphasizes the importance of a multidisciplinary approach in diagnostically challenging cases.
